# Identification of Tillering Node Proteins Differentially Accumulated in Barley Recombinant Inbred Lines with Different Juvenile Growth Habits

**DOI:** 10.3390/ijms130810410

**Published:** 2012-08-21

**Authors:** Anetta Kuczyńska, Arkadiusz Kosmala, Maria Surma, Tadeusz Adamski

**Affiliations:** Institute of Plant Genetics, Polish Academy of Sciences, Strzeszynska Str. 34, Poznan 60-479, Poland; E-Mails: akos@igr.poznan.pl (A.K.); msur@igr.poznan.pl (M.S.); tada@igr.poznan.pl (T.A.)

**Keywords:** spring barley, dwarfing gene, *denso*, 2-D electrophoresis, mass spectrometry, protein abundance

## Abstract

Barley (*Hordeum vulgare* L.) is an important cereal crop grown for both the feed and malting industries. The allelic dwarfing gene *sdw1/denso* has been used throughout the world to develop commercial barley varieties. Proteomic analysis offers a new approach to identify a broad spectrum of genes that are expressed in the living system. Two-dimensional electrophoresis and mass spectrometry were applied to investigate changes in protein abundance associated with different juvenile growth habit as effect of the *denso* locus in barley homozygous lines derived from a Maresi × Pomo cross combination. A total of 31 protein spots were revealed that demonstrate quantitative differences in protein abundance between the analyzed plants with different juvenile growth habit, and these protein spots were selected to be identified by mass spectrometry. Identification was successful for 27 spots, and functional annotations of proteins revealed that most of them are involved in metabolism and disease/defense-related processes. Functions of the identified proteins and their probable influence on the growth habit in barley are discussed.

## 1. Introduction

Semidwarf genes have been extensively explored in barley breeding programs to reduce plant height and improve the resistance to lodging. Successful use of a dwarfing gene is critical for developing dwarf cultivars [1,2]. In barley, more than 30 types of dwarfs or semidwarfs have been found. However, only a few of them have successfully been used in barley breeding programs. The dwarfing gene *uzu* on the chromosome 3HL has widely been used for barley breeding in Asia [3]. The dwarfing gene *sdw1* (previously named as *sdw*) and *denso* (which has been shown to be allelic to the *sdw1*) have been used for feed barley breeding in North America and Australia [4], and in malting barley breeding in Europe [5], respectively. Recessive alleles at the *denso* locus confer prostrate and dominant alleles-erect growth habit at the juvenile stage, providing an effective morphological marker of this gene. *Denso* dwarfing gene (= *sdw1*) has been localized on the long arm of chromosome 3H [6]. The *sdw1/denso* gene in barley is most likely the ortholog of *sd1* in rice. Comparative genomic analysis revealed that the *sdw1/denso* gene was located in the syntenic region of the rice semidwarf gene *sd1* on chromosome 1. The *sd1* gene encodes a gibberellic acid (GA)-20 oxidase enzyme that controls a step in gibberellin biosynthetic pathway. The barley and rice genes showed a similar gene structure and both share 88.3% genomic sequence similarity and 89% amino acid sequence identity. A single nucleotide mutation was identified in intron 2 and single nucleotide polymorphism (SNP) marker was mapped to chromosome 3H. Quantitative trait locus analysis revealed that plant height cosegregated with this SNP [7].

Influence of the *denso* locus on several agronomic and physiological characteristics was observed in numerous studies. Some QTLs for heading date, grain yield, thousand grain weight and plant height were localized in the *denso* region [8,9]. In addition to reduced plant height, semidwarf plants were observed to have an increased time to heading, late maturity, decreased thousand grain weight and a high level of beta-glucan [10]. There are some studies which have identified QTL for disease resistance as being associated with *sdw1* in particular, and dwarfing genes in general, through their influence on the GA signal transduction pathway [11,12]. Thus, many different QTLs mapped at the same position as the *denso* locus may indicate a pleiotropy of this gene or a tight linkage between genes conditioning the observed traits [13,14]. In practice, it is difficult to distinguish between pleiotropy and tight linkage [15]; however, it is possible to assess the relative contribution of pleiotropy and linkage disequilibrium in the control of the characters.

Anatomical effects of the *denso* gene were studied by Kuczynska and Wyka [14]. Their observations showed a coordinated dwarfing effect of the *denso* locus on cellular, tissue and whole organ level. Leaves of plants having the *denso* gene were smaller and this was reflected by smaller dimensions of some categories of epidermal cells, indicating a restriction to cellular growth. Since fully mature leaves were sampled and blade expansion had at that time terminated, observed differences could not have been caused by differences in developmental timing [13] and were rather due to gene effect on cell production rate, cellular growth, or both.

The classical approach for separating protein mixtures is 2-dimensional gel electrophoresis (2D GE), in conjunction with mass spectrometry (MS) for protein identification [16]. Proteomics has become one of the main tools in genetical genomics approaches, as shown in previous studies on different crops, where variation in protein abundance was used to map loci on the genome controlling its expression [17,18]. Proteome analysis has been used to assess natural variation among potato genotypes [19], Arabidopsis ecotypes [20] and barley cultivars differing in malting quality [21]. Segregation of protein spots on 2D gels in a doubled haploid population was used to find markers for anther culturability in barley [22]. A large genetic variability was revealed at the proteome accumulation level, which raised the possibility to predict phenotypic performance on the basis of gene product variability. This approach yielded limited results, but could be re-created by extensive identification of proteins now allowed by mass spectrometry. The dissection of the genetic basis of the variation of individual protein amounts is a very powerful tool to select “candidate” proteins, physiologically relevant for a given phenotypical trait.

In barley proteome studies, several authors mainly focused either on a more descriptive overview of occurring proteins [23–25] or they investigated the changes in protein synthesis during seed development [26]. Süle *et al.* [27] investigated the influence of short-term heat stress on the protein accumulation levels in barley. They used heat-tolerant and heat-susceptible cultivars and attempted to analyze the differentially displayed proteins after heat-shock treatment of both cultivars, in order to identify proteins responsible for heat tolerance. Such proteins are thought to be potential markers for heat tolerance of barley cultivars in breeding programs.

The comparison of 2-D protein profiles in different juvenile growth habits as an effect of the *denso* locus in barley recombinant inbred lines, and further MS analyses of differentially accumulated proteins, could be an efficient way for the identification of significant proteins involved in dwarfing effects on plant.

The aim of the studies was to identify proteins involved in barley semi-dwarfness. Comprehensive analysis at the physiological and proteomic levels included: (1) the selection of plants in different juvenile growth habit, (2) the analyses of protein accumulation profiles using 2-D electrophoresis, and (3) MS identification of proteins which were differentially accumulated between the selected plants.

## 2. Results and Discussion

**Figure 1:**
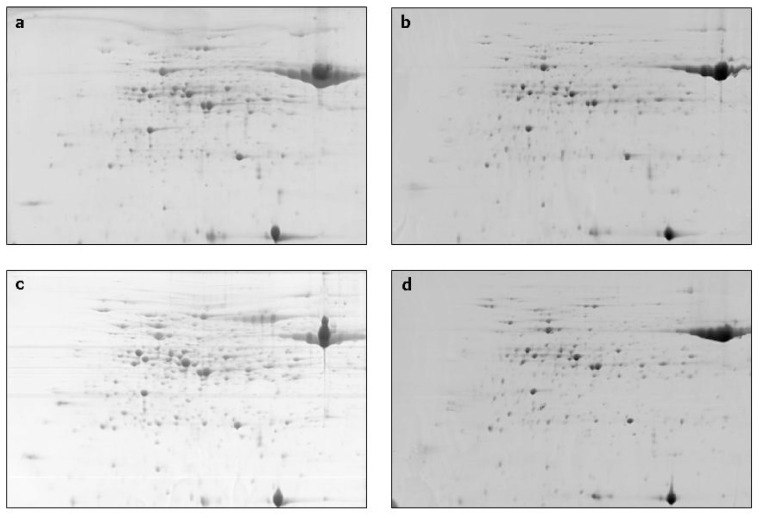
Examples of single replicates of 2-DE gels for prostrate growth habit at stage 1.3 (**a**) erect growth habit at stage 1.3; (**b**) prostrate growth habit at stage 3; (**c**) and erect growth habit at stage 3; (**d**) *Hordeum vulgare* lines. These “raw” images are not suitable to reveal the protein accumulation level, as normally the normalized volumes of spots are used for protein quantification and comparisons between gels (see in the manuscript text).

All the 2-D patterns within pH 4–7 range were shown to be well-resolved protein maps (Figure 1).

**Figure 2:**
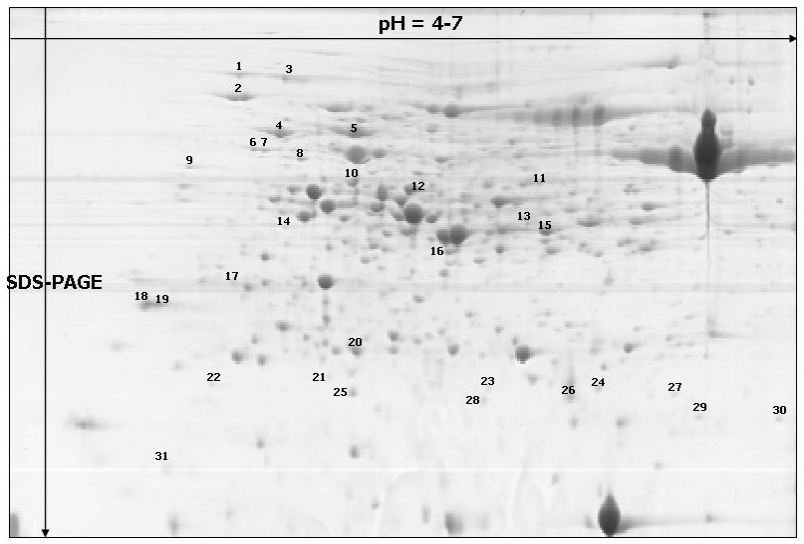
A representative 2-D protein map (based on a “raw” single replicate gel) obtained for the prostrate and erect growth habit at stage 3 barley lines. Thirty one differentially accumulated proteins are numbered on the gel.

Only the spots which were detected within two replicate gels were included in the analyses. Based on the statistical analyses of the results obtained, proper protein spots (31) were selected for further identification (Figure 2).

The proteins with the highest Multidimensional Protein Identification Technology (MudPIT) scores were selected and presented in Table 1.

**Figure 3:**
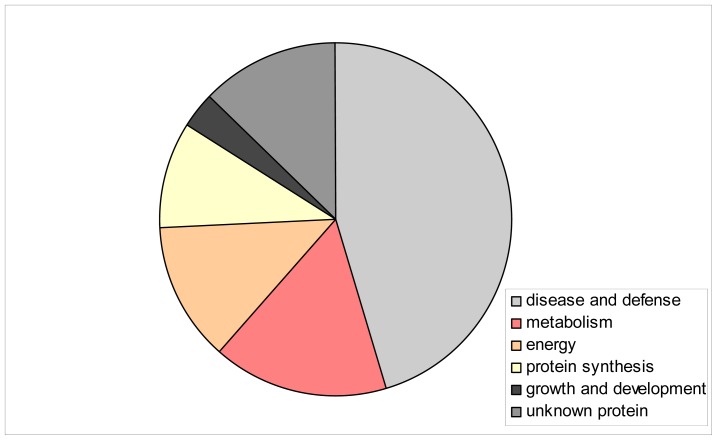
Functional classification of 31 protein spots detected in the analyzed barley recombinant inbred lines. A complete list of identified proteins is provided in Table 1.

The sequence of homologs of the identified proteins is shown as Supplementary results (Figure S1). Finally, it was possible to identify 27 out of 31 protein spots. Their functional annotations revealed that most proteins are involved in disease/defense-related processes and metabolism (Table 1, Figure 3). This classification was made according to the suggestion Witzel *et al.* [18]. Nearly 50% of the differently accumulated proteins appeared to be defense- and disease-related proteins. This may be a result of co-segregating genes involved in plant disease resistance with *sdw1* gene, because many QTLs, *inter alia* QTLs for disease resistance, were found to be associated with the *sdw1* gene [11,12].

Protein spots nos. 1, 2 and 3 were identified as heat-shock proteins (HSPs). In barley lines with prostrate growth habit, a significantly higher HSPs abundance was detected at the tillering stage, compared to the three-leaves stage. HSPs are associated with protein folding, protein translocation across membranes, assembly of oligomeric proteins, modulation of receptor activities, mRNA protection, prevention of enzyme—especially photosynthesizing—denaturation and their stress-induced aggregation, and with post-stress ubiquitin and chaperonin-aided repair. Based on these functions, HSPs have been termed “molecular chaperones” [28]. Apart from being synthesized as heat shock protein, HSPs are also accumulated in plants in response to a large number of other stress factors such as arsenite, ethanol, heavy metals, drought, light, wounding, salinity, chilling, and anoxic conditions [29].

In the present studies, protein spots, nos. 4, 5, 6, 7, 11, 14 and 29 were identified as proteins with homology to a large RuBisCO subunit-binding protein from *Triticum aestivum* or *Secale cereale* (Table 1). Rubisco is a very abundant bifunctional oligomer chloroplast enzyme which catalyzes photosynthetic carboxylation or oxygenation in plant leaves [30]. In barley plants of prostrate juvenile growth habit, an increased abundance of that enzyme was detected at the tillering stage for the majority of spots (nos. 4, 5, 6, 7, 11 and 14). In the case of protein spot no. 29, a higher protein abundance was observed at the seedling stage. In lines with erect growth habit (with exception of spots nos. 6, 7, 11), a higher accumulation level of RuBisCO large subunit-binding protein was noticed at the tillering stage. It is proposed that, under various growth conditions, part of the investment in Rubisco may be viewed as a nitrogen store, bringing additional marginal advantages with respect to photosynthetic rate and water use efficiency. A change in the rate of photosynthesis did not automatically translate into a change in the growth rate. Several factors were identified, which contribute to this buffering of growth against a changed photosynthetic rate [31].

Protein spot no. 12 was identified as chloroplast translational elongation factor. It is an essential component for protein synthesis that functions by binding aminoacylated tRNAs to the ribosome-mRNA complex. Protein spots, nos. 18 and 19 were identified as nucleic acid-binding proteins cp31BHv. These proteins significantly increased their abundance in barley plants of both growth habits at the later stage.

In barley with the prostrate growth habit, we observed a higher level of the protein derived from spot no. 22 at the three-leaves stage. During tillering, the protein accumulation level decreased and was as much as twice lower, compared to the level observed at the corresponding stage in erect growth habit lines. It was identified as ES2A protein (gibberellic acid inducible protein). Gibberellic acid (GA) is an important signaling molecule that participates in many aspects of plant growth and development [32]. While the importance of this hormone is clear, the transcriptional regulatory networks involved are still being characterized. Bioactive GAs are plant hormones that promote uniform growth through cell elongation. GAs represent a large group of cyclic diterpene compounds that promote stem elongation. Mutants in GA synthesis or signaling show dwarf phenotypes [33]. In fact, mutations in GA-related genes are responsible for the semi-dwarf habit. Fundamental research has revealed that most of the dwarfing genes were involved in the GA biosynthetic and signal transduction pathway in cereals. It is well known that the *sdw1/denso* mutants reduced plant height and were sensitive to gibberellic acid [34]. The *sdw1/denso* in barley is most likely the ortholog gene of the *sd1* in rice which carries the mutation in the gene (*Os20ox2*), encoding an oxidase enzyme (*GA20ox-2*) involved in gibberellin biosynthesis [35,36]. Although the gene function of GA20-oxidase in barley is uncertain, their ortholog genes in rice have been studied extensively. The *OsGA20ox2* (*sd1*) gene controls the step from GA_53_ to GA_44_, resulting in the levels of GA_44_, GA_19_, GA_20_, GA_1_ and GA_29_ [37,38]. Therefore, the level of GA_1_ is reduced which results in the dwarf phenotype. Based on earlier research in rice, the *sd1* orthologs gene *Hv20ox2* (barley) is predicted to control the step from GA_53_ to GA_44_. Jia *et al.* [39] indicated that high yield is associated with lower expression levels of *Hv20ox2*. They postulated that reduced expression of *Hv20ox2* in the semi-dwarfing mutants results in lower GA levels in the apical meristem, which inhibits apical growth, internode length, plant height, and promotes the development of more tillers. The temporal GA_3_-responsive expression of the ES2A transcript has been recorded only in a dwarf barley mutant [32]. In the present study, ES2A displayed an increased protein synthesis in barley lines with the prostrate growth habit at the early developmental stage—three leaves. For the same barley lines during tillering, a decrease of ES2A protein synthesis was noticed and their growth habit could be partially due to the reduced amount of this protein.

Germin-like protein 1 (GLP) was identified in a single spot: no. 26. We observed increased synthesis of the spot 26 protein in barley plants with the prostrate and erect growth habit at the three-leaves stage, and its accumulation level decreased during tillering. GLPs are encoded in plants by a gene family with proposed functions in plant development and defense. Germins are accumulated in expanding shoots of developing seedlings and young barley leaves. Remodeling of the plant cell walls during pathogen attack or abiotic stress is associated with the expression of GLPs [40]. It is the possible explanation of lower accumulation level of that protein during tillering in barley analyzed lines.

We also found a protein (spot no. 31) identified as huntingtin interacting protein K, which showed a significant increase in abundance for barley plants with both growth habits at the later stage. In-depth analysis of this protein will be necessary in order to better understand its role in relation to growth habit in barley.

In our study, in several cases, the same protein was identified in more than one spot. In fact, it was proved that not only post-translational modifications (phosphorylation, methylation and glycosylation), but also the presence of different signals and targeting sequences, *in vivo* proteolysis or *in vitro* protein degradation during sample preparation, can be a source of “new extra spots”, representing the same protein, with different positions (different molecular masses and isoelectric points) on the 2-D maps. Such multi-spot proteins often complicate the interpretation of the obtained results. The protein abundance in cells cannot be regarded as a direct reflection of the corresponding gene activity. The relationship between the transcriptome and the proteome has a complex nature and this relationship is often disturbed mainly by the post-transcriptional steps of gene expression, involving the level of proteolysis [41]. However, for the same reasons, the proteome seems to be a better indicator of cell metabolism, compared to the transcriptome.

## 3. Experimental Section

Material for the study included 270 spring barley (*Hordeum vulgare* L.) derived from the Maresi × Pomo (MP) hybrid: 197 lines obtained by the single seed descent (SSD) technique (F_6/8_) and 73 doubled-haploid lines (DH) produced by the *Hordeum bulbosum* method (F1DH) [42]. Out of 270 studied lines, 130 carried the semi-dwarfing *denso* gene. Maresi is a two-rowed, hulled, brewing cultivar, whereas Pomo is a six-rowed, hulled, fodder cultivar. The segregation in locus *denso* in both SSD and DH populations was in the ratio of 1:1, and we decided to analyze DH and SSD lines altogether.

Plant material for proteomic analyses was randomly collected from experimental plots at the Institute of Plant Genetics, Polish Academy of Sciences, Poznan, Poland. The experiment was established in a complete blocks design in two replications on plots of 2 m^2^, with a row spacing of 20 cm. Sampling was conducted at two developmental stages: three leaves (stage 1.3 according to Feekes scale) and tillering (3 on Feekes scale) [43]. In each stage, leaves of plants of prostrate and erect growth habit were bulked into two samples. Thus, four variants of plant samples, each one containing all lines in two replicates, were analyzed: (*i*) prostrate growth habit at the stage of three leaves, (*ii*) prostrate growth habit during tillering, (*iii*) erect growth habit at the stage 1.3 of three leaves, (*iv*) erect growth habit at the stage of tillering.

The protocol for proteomic research performed herein, including two-dimensional electrophoresis to analyze differences in protein accumulation levels between barley lines and mass spectrometry to identify differentially accumulated proteins, was the same as that described in detail by Kosmala *et al.* [44] and Bocian *et al.* [45]. Protein extraction was performed according to the method described by Hurkman and Tanaka [46], and protein concentration was determined by using the 2-D Quant Kit (GE Healthcare, Buckinghamshire, UK). In the first dimension, isoelectrofocusing (IEF), 24 cm Immobiline DryStrip gels with linear pH range 4–7 were used to focus the aliquots of proteins (0.5 mg) extracted from 100 mg of barley tissues. In the second dimension (sodium dodecyl sulfate-polyacrylamide gel electrophoresis), the proteins were separated using 13% polyacrylamide gels (1.5 × 255 × 196 mm). Following electrophoresis, the gels were stained with colloidal Coomassie Brilliant Blue G-250, using the modified method of Neuhoff *et al.* [47]. Total separated protein spots on the gels were scanned by ImageScanner III (GE Healthcare) and subjected to LabScan 6.0 program (GE Healthcare) processing. Spot detection and image analyses (normalization, spot matching and protein quantification) were performed with Image Master 2-D Platinum software (GE Healthcare). To compensate for subtle differences in sample loading, gel staining and destaining, the abundance of each protein spot was normalized as a relative volume (% vol). Percent volume of each spot was automatically calculated by Image Master software as a ratio of the volume of particular spot to the total volume of all the spots present on the gel. The extraction procedure and electrophoretic separation were performed twice; thereafter, % vol for the spots from the two replicated gels was used to calculate means and standard deviations. The spots with at least 2-fold differences (*p* ≤ 0.05) in protein abundance (quantitative differences between gels) between at least two different plant variants, were subjected to MS analyses and protein identification.

Protein spots were excised from the gel and analyzed by liquid chromatography, coupled to the mass spectrometer in the Laboratory of Mass Spectrometry, Institute of Biochemistry and Biophysics, Polish Academy of Sciences, Warsaw, Poland. Samples were concentrated and desalted on a RP-C18 trap-column (Waters: Milford, MA, USA), and further peptide separation was conducted on a nano-Ultra Performance Liquid Chromatography (UPLC) RP-C18 column (Waters, BEH130 C18 column, 75 μm i.d., 250 mm long) of a nanoACQUITY UPLC system, using a linear acetonitrile gradient in the range of 5%–30% in 45 min. Column outlet was directly coupled to the Electrospray ionization (ESI) ion source of Orbitrap type mass spectrometer (Thermo), working in the regime of data-dependent MS to MS/MS switch. An electrospray voltage of 2 kV was used. Raw data files were pre-processed with Mascot Distiller software (version 2.3.2.0, MatrixScience: London, UK). The peptide masses obtained and fragmentation spectra were matched to the National Center Biotechnology Information (NCBI) non-redundant database with a *Viridiplantae* filter (884942 sequences), using the Mascot search engine (Mascot Daemon version 2.3.0, Mascot Server version 2.2.03, MatrixScience: London, UK). The following search parameters were applied: enzyme specificity was set to trypsin, peptide mass tolerance to ± 40 ppm and fragment mass tolerance to ± 0.8 Da. The protein mass was left as unrestricted, and mass values as monoisotopic with one missed cleavage being allowed. Alkylation of cysteine by carbamidomethylation as fixed, and oxidation of methionine was set as a variable modification. Protein identification was performed using the Mascot search probability-based Mowse score. Ion score was −10 log(*p*), where *p* was the probability that the observed match was a random event. Mascot defined threshold which indicated identity or extensive homology (*p* < 0.05) was 40 or less, therefore ion score 40 was taken as a threshold for analysis. When the protein was selected as the predicted protein, the sequence of the predicted protein was blasted using blastp algorithm. The protein with the highest score was then selected as the functional homolog of “the predicted protein” (presented in Table 1).

## 4. Conclusions

2-DE coupled with MS led to the identification of various proteins, which may be involved in the phenotypic effect of the *denso* locus in barley. Identified proteins were revealed to show quantitative differences in their abundance between the analyzed plants with prostrate and erect growth habit at the juvenile stage. Most of these proteins are involved in metabolism and disease/defense-related processes. ES2A protein (expression of the gene coding this protein is GA_3_-responsive and GA-related genes are responsible for the semi-dwarf habit) displayed an increased protein synthesis in barley lines with the prostrate growth habit at the three-leaves stage. For the same barley lines, a decrease in ES2A protein synthesis was noticed during tillering and their growth habit could be partially due to the reduced amount of this protein.

This experiment is the first proteome analysis on tillering node proteins in different juvenile growth habits as an effect of the *denso* locus in barley recombinant inbred lines. In addition to providing new information, the present study offers opportunities to pursue analysis with *sdw1/denso* associated physiological characters, determining yield, to effectively use this gene in a breeding program.

## Supplementary Materials



## Figures and Tables

**Table 1:** The results of MS analysis performed on proteins differentially accumulated between distinct juvenile growth habits of barley.

Spot no. a	% Vol				Accession f	Identified protein g	Score h	Coverage (%) i	Classification

	1.3 p b	3 p c	1.3 e d	3 e e					
1 *	0.1203	0.4296	0.1818	0.1977	CAA82945	Heat-shock protein, *S. cereal*	3526	47	disease and defense
2 *	0.5020	1.0492	0.5464	0.7669	ACT65562	70 kDa heat shock protein, *T. aestivum*	2581	38	disease and defense
3 *	0.3994	0.5682	0.6887	0.8340	CAA47948	Heat shock protein 70, *O. sativa*	5974	58	disease and defense
4	0.1899	0.5595	0.4675	1.0772	PO8823	RuBisCO large subunit-binding protein subunit alfa, chloroplastic (60 kDa chaperonin subunit alfa), *T.aestivum*	15416	58	disease and defense
5	0.5488	1.1695	0.8930	1.3915	Q43831	RuBisCO large subunit-binding protein subunit beta, chloroplastic (60 kDa chaperonin subunit beta), *S. cereal*	11239	65	disease and defense
6	0.0487	0.1235	0.1173	0.1244	Q43831	RuBisCO large subunit-binding protein subunit beta, chloroplastic (60 kDa chaperonin subunit beta), *S. cereal*	4325	55	disease and defense
7	0.0577	0.0970	0.1091	0.1279	Q43831	RuBisCO large subunit-binding protein subunit beta, chloroplastic (60 kDa chaperonin subunit beta), *S. cereal*	2326	37	disease and defense
8 *	0.0474	0.0878	0.0676	0.1043	Unknown	Unknown	2492	28	-
9	0.0243	0.0706	0.0465	0.0469	NP_001056601	Os06go114000–hypothetical protein similar to 60 kDa chaperonin (Protein Cpn60), *O. sativa*	2136	20	disease and defense
10	0.2498	0.3715	0.1788	0.2889	Q40073	Ribulase bisphosphate carboxylase/oxygenase activase A, chloroplastic; (RuBisCO activase A), *H. vulgare*	4802	49	metabolism
11	0.0359	0.0984	0.0654	0.0511	Q43831	RuBisCO large subunit-binding protein subunit beta, chloroplastic; (60 kDa chaperonin subunit beta), *S. cereale*	1048	26	disease and defense
12	0.2444	0.5373	0.2588	0.5118	AAF15312	Chloroplast translational elongation factor Tu, *O. sativa*	1269	22	protein synthesis
13	0.0888	0.1296	0.0531	0.0681	AAF71272	Ribulose bisphosphate carboxylase activase B, *T. aestivum*	1735	29	metabolism
14	0.0280	0.0582	0.0183	0.0477	Q43831	RuBisCO large subunit-binding protein subunit beta, chloroplastic (60 kDa chaperonin subunit beta), *S. cereale*	1075	19	disease and defense
15 *	0.6958	1.0498	0.5114	0.5135	Unknown	Unknown	3733	44	-
16	0.0787	0.1996	0.0753	0.0834	CAD30025	Ferredoxin-NADP (H) oxidoreductase B, *T. aestivum*	1976	33	energy
17 *	0.0726	0.1294	0.0472	0.0697	Unknown	Unknown	1011	36	-
18	0.1338	0.2438	0.0697	0.2215	CAA11893	cp31BHv (nucleic acid-binding protein), *H. vulgare*	1081	29	metabolism
19	0.1001	0.2228	0.1023	0.1851	CAA11893	cp31BHv (nucleic acid-binding protein), *H. vulgare*	1170	36	metabolism
20 *	0.1431	0.2969	0.1893	0.2896	ACG41110.1	chaperonin, *Z. mays*	3114	85	disease and defense
21 *	0.1516	0.0367	0.0957	0.0683	Unknown	Unknown	348	29	-
22	0.1540	0.0357	0.0207	0.0720	CAA55976	ES2A (gibberellic acid (GA3) inducible), *H. vulgare*	987	74	growth and development
23 *	0.0456	0.0386	0.0141	0.0352	ACG41110.1	Chaperonin, *Z. mays*	230	22	disease and defense
24 *	0.0321	0.0706	0.0309	0.0465	BAD22518.1	glycolipid transfer protein-like, *O. sativa*	113	14	energy
25 *	0.0974	0.1335	0.0580	0.0311	YP_874661.1	ribulose 1,5-bisphosphate carboxylase/oxygenase large subunit, *H. vulgare*	708	10	energy
26	0.5010	0.4965	0.7231	0.3582	BAA74702	Germin-like protein 1, *O. sativa*	2249	15	disease and defense
27	0.0109	0.0421	0.0114	0.0260	AAT40531.1	ATP synthase D chain, mitochondrial, putative, *S. demissum*	161	20	metabolism
28	0.1267	0.0516	0.0519	0.0479	AAZ95171	Eukaryotic translation initiation factor 5A1	447	26	protein synthesis
29	0.1315	0.0455	0.0455	0.0695	Q43831	RuBisCO large subunit-binding protein subunit beta, chloroplastic; (60 kDa chaperonin subunit beta), *S. cereale*	1069	14	disease and defense
30 *	0.0650	0.0589	0.0351	0.0929	AAP44537.1	cyclophilin-like protein, *T. aestivum*	567	30	protein synthesis
31	0.0373	0.1243	0.0530	0.1459	ACG36699	Huntingtin interacting protein K, *Z. mays*	142	22	energy

a Spot numbering was the same as in Figure 2.

* the protein identity was established after using the blastp algorithm as described in the text;

b The mean of spot relative volumes (% vol) for plants represent prostrate growth habit at stage 1.3 according to Feekes scale;

c The mean of spot relative volumes (% vol) for plants represent prostrate growth habit at stage 3 according to Feekes scale;

d The mean of spot relative volumes (% vol) for plants represent erect growth habit at stage 1.3 according to Feekes scale;

e The mean of spot relative volumes (% vol) for plants represent erect growth habit at stage 3 according to Feekes scale;

f Database accession (according to NCBInr) of a homologous protein;

g Homologous protein and organism from which it originates;

h Mascot MudPIT (Multidimensional Protein Identification Technology) score;

i Amino acid sequence coverage for the identified proteins. The full sequence of the homologs of the identified proteins is shown in Supplementary results (Figure S1).
