# Supplementary material for: Identification of Tillering Node Proteins Differentially Accumulated in Barley Recombinant Inbred Lines with Different Juvenile Growth Habits
Source: Int J Mol Sci. 2012 Aug 21;13(8):10410–23. doi: 10.3390/ijms130810410 (PMC3431868; doi:10.3390/ijms130810410)
Supplement: Supplementary file 1 [file ijms-13-10410-s001.pdf]

Supplementary Material

Figure S1. The sequence of the homologues of the identified barley proteins. (Matched peptides shown in Bold Red. When the protein was selected as the predicted protein, the sequence of the predicted protein was blasted using blastp algorithm. The protein with the highest score was then selected as the functional homolog of "the predicted protein" and it was also presented in Table 1).

Spot 1. Predicted protein; gi|326517328; BAK00031; Score: 3526; [Hordeum vulgare subsp. vulgare].

1 MAPALSRTLG PSSVAALRPS PSRGLPRAAL APQGRRPAGA RGVRWEAGRE
51 RLVGARCASA VAEKTAGEEE AAGEEFEYQA EVSR**LMDLIV** **HSLYSHKEVF**
101 LR**ELVSNASD** **ALDKLRFLSV** **TDPSVLADGG** **DMEIRIKPDP** **DAGTITITDS**
151 **GIGMTKDELK** **DCLGTIAQSG** **TSK**FLKALKE NKELGADNGL IGQFGVGFYS
201 AFLVAEKVVV STKSPKTDK**Q** **YIWEAEANSS** **SYVIR**EETDP EKMLTR**GTQI**
251 **TLFLREDDKY** **EFADPTR**IQG LVKNYSQFVS FPIFTWQEKS R**TVEVEEEES**
301 **KETEETAEGE** **K**EKKKKTITE KYWDWELANE TKPIWMRNPK **EVEETEYNEF**
351 **YKK**AFNEFLD PLAHAHFTTE GEVEFR**SVLY** **IPGMAPLSNE** **EIMNPK**TKNI
401 RLYVKRVFIS DDFDGELFPR YLSFVK**GVVD** **SNDLPLNVSR** EILQESRIVR
451 IMRKRLVRK**T** **FDMIQDIADK** **DEK**EDYKKFW ESFGKFMK**LG** **CIEDSGNQKR**
501 LAPLLRFYSS K**NETDLISLD** **QYVENMPETQ** **KAIYYIATDS** **LQSAK**TAPFL
551 EKLLQKDIEV LYLIEPIDEV AIQNLQTYKE K**KFVDISKED** **LELGDEDEDK**
601 **EESK**QEYTLL CDWIKQQLGD KVAKVQISK**R** **LSSSPCVLVS** **GKFGWSANME**
651 **RLMKAQTLGD** **TSSLEFMR**GR **RIFEINPEHP** **IVK**DLSAACK NEPESTEAKR
701 AVELLYETAL ISSGYTPESP AELGGK**IYEM** **MTIALGGR**WG R**SGTEEAETN**
751 **VNDSSEAVVT** **EVVEPSEVRT** **ENENDPWRD**

Blast

heat-shock protein; CAA82945; [Secale cereale].

Spot 2. Predicted protein; gi|326492960; BAJ90336; Score: 2581;[Hordeum vulgare subsp. vulgare].

1 MSIFTSQVSA MASASPSSSL FVSRRRPAVM PLQMRVARGG RPRGLTMRVT
51 CEK**VVGIDLG** **TTNSAVAAME** **GGKPTVITNA** **EGQR**TTPSVV AYTKGGERLV
101 GQIAK**RQAVV** **NPENTFFSVK** **R**FIGR**KMAEV** **DDEAK**QVSYN VVRDENGNVK
151 **LDCPAIGKQF** **AAEEISAQVL** **R**KLVDDASKF LNDKITK**AVV** **TVPAYFNDSQ**
201 **R**TATKDAGR**I** **AGLEVLRIIN** **EPTAASLAYG** **FEK**KNNETIL VFDLGGGTFD
251 VSVLEVGDGV FEVLSTSGDT HLGGDDFDKK **IVDWLASTFK** **NDEGIDLLKD**
301 **K**QALQRLTEA AEKAKMELST LTQANISLPF ITATADGPK**H** **IEATLSRAKF**
351 **EELCSNLIDR** **LKTPVNNALK** DAKLSISNLD EVILVGGSTR **IPSVQETVR**K
401 ITGKDPNVTV NPDEVVSLGA AVQGGVLAGD VKDVVLLDVT PLSIGLETLG
451 GVMTKIIPRN TTLPTSKSEV FSTAADGQTS VEINVLQGER EFVRDNKSLG
501 SFRLDGIPPA PRGVPQIEVK FDIDANGILS VAAVDKGTGK **KQDITITGAS**
551 **TLPKDEVERM** **VEEADKFAQE** **DKEK**RDAIDT K**NQADSVVYQ** **TEKQLKELGD**
601 **KVPAPVKEK**V DVK**LQELKDA** **IAGGSTQSMK** TSMEALNQEV MQIGQAMYNQ
651 TSAGGAGSTD AEAEPTPSAG STSSGK**GPND** **GDVIDADFTD** **SN**

Blast

70 kDa heat shock protein; ACT65562.1; [Triticum aestivum].

Spot 3. Predicted protein; gi|326506132; BAJ91305; Score: 5974;[Hordeum vulgare subsp. vulgare].

```
  1 MAAKGDGPAI GIDLGTTYSC VGVWQHDRVE IIANDQGNRT TPSYVAFTDS
 51 ERLIGDAAKN QVAMNPINTV FDAKRLIGRR FTDSTVQSDI KLWPFKVVAG
101 PGDKPMINVQ YKGEEKQFAA EEISSMVLIK MREIAEAFLG STVKNAVVTV
151 PAYFNDSQRQ ATKDAGVIAG INVLRIINEP TAAAIAYGLD KKASSVGEKN
201 VLIFDLGGGT FDVSLLTIEE GIFEVKATAG DTHLGGEDFD NRMVNHFVQE
251 FKRKNKKDIS GNPRALRRLR TSCERAKRTL SSTAQTTIEI DSLFEGIDFY
301 STITRARFEE MNMDLFRKCM EPVEKCLRDA KMDKSTVHDV VLVGGSTRIP
351 KVQQLLQDFF NGKELCKSIN PDEAVAYGAA VQAAILSGEG NEKVQDLLLL
401 DVTPLSLGLE TAGGVMTVLI PRNTTIPTKK EQVFSTYSDN QPGVLIQVYE
451 GERTRTRDNN LLGKFELSGI PPAPRGVPQI TVCFDIDANG ILNVSAEDKT
501 TGQKNKITIT NDKGRLSKEE IEKMVQEAEK YKSEDEEHKK KVESKNALEN
551 YSYNMRNTIK DEKIASKLPA DDKKKIEEAI DAAIQWLDTN QLAEADEFED
601 KMKELEALCN PIIAKMYQGA GADMEGGMDD DTPAASGGPG PKIEEVD
```

Blast

heat shock protein 70; CAA47948.2; [Oryza sativa Indica Group].

Spot 4. RuBisCO large subunit-binding protein subunit alpha, chloroplastic (60 kDa chaperonin subunit alpha); gi|134102; P08823; Score: 15416; [Triticum aestivum].

```
  1 GADAKEIAFD QKSRAALQAG VEKLANAVGV TLGPRGRNVV LDEYGNPKVV
 51 NDGVTIARAI ELANPMENAG AALIREVASK TNDSAGDGTT TACVLAREII
101 KLGILSVTSG ANPVSLKKGI DKTVQGLIEE LERKARPVKG SGDIKAVASI
151 SAGNDELIGA MIADAIDKVG PDGVLSIESS SSFETTVDVE EGMEIDRGYI
201 SPQFVTNLEK SIVEFENARV LITDQKITSI KEIIPLLEQT TQLRCPLFIV
251 AEDITGEALA TLVVNKLRGI INVAAIKAPS FGERRKAVLQ DIAIVTGAEY
301 LAKDLGLLVE NATVDQLGTA RKITIHQTTT TLIADAASKD EIQARVAQLK
351 KELSETDSIY DSEKLAERIA KLSGGVAVIK VGATTETELE DRQLRIEDAK
401 NATFAAIEEG IVPGGGAAYV HLSTYVPAIK ETIEDHDERL GADIIQKALQ
451 APASLIANNA GVEGEVVIEK IKESEWEMGY NAMTDKYENL IESGVIDPAK
501 VTRCALQNAA SVSGMVLTTQ AIVVEKPKPK PKVAEPAEGQ LSV
```

Spot 5. RuBisCO large subunit-binding protein subunit beta, chloroplastic (60 kDa chaperonin subunit beta); gi|2493650; Q43831; Score: 11239; [Secale cereale].

```
  1 PQIVNDGVTV AREVELEDPV ENIGAKLVRQ AAAKTNDLAG DGTTTSVVLA
 51 QGLIAEGVKV IAAGANPVQI TRGIEKTAKA LVLELKKMSK EVEDSELADV
101 AAVSAGNNYE IGNMIAEAMS KVGRKGVVTL EEGRSSENNL YVVEGMQFER
151 GYISPYFVTD SEKMTTEYEN CKLLLVDKKI TNARDLINVL EEAIRGQYPI
201 LIIAEDIEQE ALATLVVNKL RGSLKICAIK APGFGERKTQ YLDDIAILTG
251 GTVIRDEVGL TLDKADNTVL GTAAKVVLTK ESTTIVGDGS TQEEVTKRVA
301 QIKNLIEAAE QDYEKEKLNE RIAKLAGGVA VIQVGAQTET ELKEKKLRVE
351 DALNATKAAV EEGIVVGGGC TLLRLAAKVD AIKDTLENDE QKVGAEIVRR
401 ALCYPLKLIA KNAGVNGSVV TEKVLSNDNF KFGYNAATGQ YEDLMAAGII
451 DPTKVVRCCL EHAASVAKTF LTSDVVVVEI KEPEAAPLAN PMDNSGFGY
```

Spot 6. RuBisCO large subunit-binding protein subunit beta, chloroplastic (60 kDa chaperonin subunit beta); gi|2493650; Q43831; Score: 4325; [Secale cereale].

```
  1 PQIVNDGVTV AREVELEDPV ENIGAKLVRQ AAAKTNDLAG DGTTTSVVLA
 51 QGLIAEGVKV IAAGANPVQI TRGIEKTAKA LVLELKKMSK EVEDSELADV
101 AAVSAGNNYE IGNMIAEAMS KVGRKGVVTL EEGRSSENNL YVVEGMQFER
151 GYISPYFVTD SEKMTTEYEN CKLLLVDKKI TNARDLINVL EEAIRGQYPI
201 LIIAEDIEQE ALATLVVNKL RGSLKICAIK APGFGERKTQ YLDDIAILTG
251 GTVIRDEVGL TLDKADNTVL GTAAKVVLTK ESTTIVGDGS TQEEVTKRVA
301 QIKNLIEAAE QDYEKEKLNE RIAKLAGGVA VIQVGAQTET ELKEKKLRVE
351 DALNATKAAV EEGIVVGGGC TLLRLAAKVD AIKDTLENDE QKVGAEIVRR
401 ALCYPLKLIA KNAGVNGSVV TEKVLSNDNF KFGYNAATGQ YEDLMAAGII
451 DPTKVVRCCL EHAASVAKTF LTSDVVVVEI KEPEAAPLAN PMDNSGFGY
```

Spot 7. RuBisCO large subunit-binding protein subunit beta, chloroplastic (60 kDa chaperonin subunit beta); gi|2493650; Q43831; Score: 2326; [Secale cereale].

```
  1 PQIVNDGVTV AREVELEDPV ENIGAKLVRQ AAAKTNDLAG DGTTTSVVLA
 51 QGLIAEGVKV IAAGANPVQI TRGIEKTAKA LVLELKKMSK EVEDSELADV
101 AAVSAGNNYE IGNMIAEAMS KVGRKGVVTL EEGRSSENNL YVVEGMQFER
151 GYISPYFVTD SEKMTTEYEN CKLLLVDKKI TNARDLINVL EEAIRGQYPI
201 LIIAEDIEQE ALATLVVNKL RGSLKICAIK APGFGERKTQ YLDDIAILTG
251 GTVIRDEVGL TLDKADNTVL GTAAKVVLTK ESTTIVGDGS TQEEVTKRVA
301 QIKNLIEAAE QDYEKEKLNE RIAKLAGGVA VIQVGAQTET ELKEKKLRVE
351 DALNATKAAV EEGIVVGGGC TLLRLAAKVD AIKDTLENDE QKVGAEIVRR
401 ALCYPLKLIA KNAGVNGSVV TEKVLSNDNF KFGYNAATGQ YEDLMAAGII
451 DPTKVVRCCL EHAASVAKTF LTSDVVVVEI KEPEAAPLAN PMDNSGFGY
```

Spot 8. Predicted protein; gi|326490439; BAJ84883; Score: 2492; [Hordeum vulgare subsp. vulgare].

```
  1 MLSVSHPHPA ASTGPRHRKP LSTAHHRRRR CTYTIAALIL PGGGGPRGSP
 51 PNGGKLILPG SGGGGGRGGG VGGGMLPRTP PPTAPPGQLY QPFHPPPSPL
101 PENYRNLDLT ERLAVLRDRM GRWYEYAPLI SSLSREGFTP ASIEEATGMS
151 GVEQNRLVVA SQVRDSLISD DFPDDLLHYF DSYGGPDLLY ELRFLNARQR
201 IVATKHTIER RLESKGVREL ARSMKDFPQR RGDEGWDAFD RHSAGDCLAY
251 ARFRLSREAI ANEDRIPELE RSLDVVETES ARARVELEVE RAIKKAAGEE
301 VEELEAKVDA RPAVPVVRLM YGEISEASIV LLLPVVKETD GVQAVDLAPR
351 RSQTDADLGI VEVDKGWARW AVLPGWAPVM AVADEAVVIE LADGRVLPWR
401 SAENERVLVV ADRKRKEVVD EGIYVLEKGG KLVVERGNKL LEEGISQAAA
451 EVVTVVRPPK DEEDIIVGDE WD
```

Blast

Any recognized protein with high homology.

Spot 9. Os06g0114000—hypothetical protein similar to 60 kDa chaperonin (Protein Cpn60); gi|115466004; NP_001056601; Score: 2136; [Oryza sativa Japonica Group].

```
  1 MASTFGATST VGLMAAPTGI VSDKKPSSLS SVSSVSVASR PRNARLQRKC
 51 NFRVKAAKEL YFNKDGSAIK KLQTGVNKLA DLVGVTLGPK GRNVVLESKY
101 GSPRIVNDGV TVAREVELED PVENIGAKLV RQAAAKTNDL AGDGTTTSVV
151 LAQGLIAEGV KVVAAGANPV QITRGIEKTA KALVEELKKL SKEVEDSELA
201 DVAAVSAGNN YEIGNMIAEA MSKVGRKGVV TLEEGRSSEN NLYVVEGMQF
251 ERGYISPYFV TDSEKMSAEY ENCKLLLVDK KITNARDLIN VLEEAIRGAY
301 PILIIAEDIE QEALATLVVN KLRGSLKIAA IKAPGFGERK TQYLDDIAIL
351 TGATVIRDEV GLSLDKADKS VLGTAAKVVL NKESTTIVGD GSTQEEVTKR
401 VAQIKNLIEA AEQEYEKEKL NERIAKLAGG VAVIQVGAQT ETELKEKKLR
451 VEDALNATKA AVEEGIVVGG GCTLLRLAAR VDAIKDNLEN DEQKVGAEIV
501 RRALSYPLKL IAKNAGVNGS VVTEKVLSND NFKFGYNAAT GQYEDLMAAG
551 IIDPTKVVRC CLEHAASVAK TFLTSDVVVV EIKEPEPAPV TNPMDNSGYG
601 Y
```

Spot 10. Ribulose bisphosphate carboxylase/oxygenase activase A, chloroplastic; (RuBisCO activase A); gi|12643756; Q40073;Score: 4802; [Hordeum vulgare subsp. vulgare].

```
  1 MAAAFSSTVG APASTPTNFL GKKLKKQVTS AVNYHGKSSK ANRFTVMAAE
 51 NIDEKRNTDK WKGLAYDISD DQQDITRGKG IVDSLFQAPT GHGTHEAVLS
101 SYEYVSQGLR KYDFDNTMGG FYIAPAFMDK LVVHLSKNFM TLPNIKIPLI
151 LGIWGGKGQG KSFQCELVFA KMGINPIMMS AGELESGNAG EPAKLIRQRY
201 REAADMIKKG KMCCLFINDL DAGAGRMGGT TQYTVNNQMV NATLMNIADA
251 PTNVQLPGMY NKRENPRVPI VVTGNDFSTL YAPLIRDGRM EKFYWAPTRD
301 DRIGVCKGIF QTDNVSDESV VKIVDTFPGQ SIDFFGALRA RVYDDEVRKW
351 VGSTGIENIG KRLVNSRDGP VTFEQPKMTV EKLLEYGHML VQEQDNVKRV
401 QLADTYMSQA ALGDANQDAM KTGSFYGKGA QQGTLPVPEG CTDQNAKNYD
451 PTARSDDGSC LYTF
```

Spot 11. RuBisCO large subunit-binding protein subunit beta, chloroplastic; (60 kDa chaperonin subunit beta); gi|2493650; Q43831; Score: 1048; [Secale cereale].

```
  1 PQIVNDGVTV AREVELEDPV ENIGAKLVRQ AAAKTNDLAG DGTTTSVVLA
 51 QGLIAEGVKV IAAGANPVQI TRGIEKTAKA LVLELKKMSK EVEDSELADV
101 AAVSAGNNYE IGNMIAEAMS KVGRKGVVTL EEGRSSENNL YVVEGMQFER
151 GYISPYFVTD SEKMTTEYEN CKLLLVDKKI TNARDLINVL EEAIRGQYPI
201 LIIAEDIEQE ALATLVVNKL RGSLKICAIK APGFGERKTQ YLDDIAILTG
251 GTVIRDEVGL TLDKADNTVL GTAAKVVLTK ESTTIVGDGS TQEEVTKRVA
301 QIKNLIEAAE QDYEKEKLNE RIAKLAGGVA VIQVGAQTET ELKEKKLRVE
351 DALNATKAAV EEGIVVGGGC TLLRLAAKVD AIKDTLENDE QKVGAEIVRR
401 ALCYPLKLIA KNAGVNGSVV TEKVLSNDNF KFGYNAATGQ YEDLMAAGII
451 DPTKVVRCCL EHAASVAKTF LTSDVVVVEI KEPEAAPLAN PMDNSGFGY
```

Spot 12. Chloroplast translational elongation factor Tu; gi|6525065; AAF15312; Score: 1269; [Oryza sativa Japonica Group].

```
  1 MASLASASAS TSLVFSTSSS KPRLGSSVGF SSPARFRRTA AAAASKGTGR
 51 RAGLLVMRAA RGKFERTKPH VNIGTIGHVD HGKTTLTAAL TMVLASVGGS
101 APKKYDEIDA APEERARGIT INTATVEYET ETRHYAHVDC PGHADYVKNM
151 ITGAAQMDGA ILVVSGADGP MPQTKEHILL AKQVGVPKIV VFLNKKDQVD
201 DEELLQLVEL EVRELLSSYE YDGDEVPIVA GSALKALENL MANPAIKRGD
251 DEWVDGIFSL IDSVDNYIPV PQRQTDLPFL LAVEDVFSIT GRGTVATGRI
301 ERGTVKVGDT VDIVGIRETR NCTVTGVEMF QKTMDDAMAG DNVGLLLRGM
351 QKDDIERGMV LAKPASITPH TKFDAVVYVL KKDEGGRHSP FFPGYRPQFY
401 MRTTDVTGNV PKIMNDKDEE AKMCMPGDRV KMVVELIQPV ACEQGMRFAI
451 PEGGKTVGAG VINTILK
```

Spot 13. Ribulose bisphosphate carboxylase activase B; gi|7960277; AAF71272; Score: 1735; [Triticum aestivum].

```
  1 MASAFSSTVG APASTPTTFL GKKVKKQAGA LNYYHGGNKI NNRVVRAMAA
 51 KKELDEGKQT DADRWKGLAY DISDDQQDIT RGKGIVDSLF QAPMGDGTHE
101 AILSSYEYIS QGLRKYDFDN TMDGLYIAPA FMDKLIVHLA KNFMTLPNIK
151 VPLILGIWGG KGQGKSFQCE LVFAKMGINP IMMSAGELES GNAGEPAKLI
201 RQRYREAADI IKKGKMCCLF INDLDAGAGR MGGTTQYTVN NQMVNATLMN
251 IADAPTNVQF PGMYNKEENP RVPIIVTGND FSTLYAPLIR DGRMEKFYWA
301 PTREDRIGVC KGIFRTDNVP DEAVVRLVDT FPGQSIDFFG ALRARVYDDE
351 VRKWVGEIGV ENISKRLVNS REGPPTFDQP KMTIEKLMEY GHMLVQEQEN
401 VKRVQLADKY LSEAALGQAN DDAMATGAFY GK
```

Spot 14. RuBisCO large subunit-binding protein subunit beta, chloroplastic (60 kDa chaperonin subunit beta); gi|2493650; Q43831; Score: 1075; [Secale cereale].

```
  1 PQIVNDGVTV AREVELEDPV ENIGAKLVRQ AAAKTNDLAG DGTTTSVVLA
 51 QGLIAEGVKV IAAGANPVQI TRGIEKTAKA LVLELKKMSK EVEDSELADV
101 AAVSAGNNYE IGNMIAEAMS KVGRKGVVTL EEGRSSENNL YVVEGMQFER
151 GYISPYFVTD SEKMTTEYEN CKLLLVDKKI TNARDLINVL EEAIRGQYPI
201 LIIAEDIEQE ALATLVVNKL RGSLKICAIK APGFGERKTQ YLDDIAILTG
251 GTVIRDEVGL TLDKADNTVL GTAAKVVLTK ESTTIVGDGS TQEEVTKRVA
301 QIKNLIEAAE QDYEKEKLNE RIAKLAGGVA VIQVGAQTET ELKEKKLRVE
351 DALNATKAAV EEGIVVGGGC TLLRLAAKVD AIKDTLENDE QKVGAEIVRR
401 ALCYPLKLIA KNAGVNGSVV TEKVLSNDNF KFGYNAATGQ YEDLMAAGII
451 DPTKVVRCCL EHAASVAKTF LTSDVVVVEI KEPEAAPLAN PMDNSGFGY
```

Spot 15. Predicted protein; gi|326523629; BAJ92985; Score: 3733; [Hordeum vulgare subsp. vulgare].

```
  1 MAFSPATTAA ATAAGAITFT SGRVASSPPS SLSSPFLPRA AGAIVARRGR
 51 AGARAASAVP VRAQAAGAAK KKVLVVNTNS GGHAVIGFYF AKALLAAGHD
101 VTLLTVGDEA SDKMKKPPFS RFSELTSAGA KTVWGDPADV GAAVGAASFD
151 VVLDNNGKDL DAVKPVADWA KAAGVGQFLF ISSAGIYLQT DEPPHVEGDA
201 VKESAGHVGV EKYIAAEFGS WASFRPQYMT GSGNNKDCEE WFFDRVVRKR
251 PVPIPGSGMQ LTNISHARDL GSMLTLAVEN PDAAAGKIFN CVSDRGVTLD
301 GLAKMCAAAA GATVEIVHYD PAAVGVDAKK AFPFRNMHFY AEPRAAKEVL
351 GWTSTTNLPE DLKERFAEYA SSGRGEKAMT FDLDDKILAA VGAAPVSVAA
401
```

Blast

Any recognized protein with high homology.

Spot 16. Ferredoxin-NADP(H)oxidoreductase; gi|20302473; CAD30025; Score: 1976; [Triticum aestivum].

```
  1 MAAQLTAALP SYSPATTKAA AGGSSPSSHF LAYPSRPRNV RNGVRAQVST
 51 TEPTAEPAPA APAKPVKISK KQDEGVVTNK YRPKEPYVGR CLLNTRLTGD
101 NAPGETWHMV FSTEGEVPYR EGQSIGVIAD GEDKNGKPHK LRLYSIASSA
151 LGDFGDSKTV SLCVKRLVYT NDAGEVVKGV CSNFLCDLKP GSEVKITGPV
201 GKKMLMPKDP NATIIMLATG TGIAPFRSFL WKMFFEEHED YKFNGLAWLF
251 LGVPTSDTLL YKEEFEKMVE IGGENFRLDF AVSREQTNAA GEKMYIQTRM
301 AEYKEELWEM LKKDNTYVYM CGLKGMEKGI DDIMVDLAAK DGIDWIDYKK
351 QLKKAEQWNV EVY
```

Spot 17. Predicted protein; gi|326500884; BAJ95108; Score: 1011; [Hordeum vulgare subsp. vulgare].

```
  1 MAATYCAYPA ASAAAAANLT RRRPQTLNSP GALPAVRKPS RQPPSFLSFR
 51 RPNAALPPLR VAGADPQIVN GEDFPPMNDL IRLYKKAFLD GNEDVVSDIE
101 KAITSMEEER SKAASQFDSI TAEITSGKNK FLRLNADLEN FRKQTEKDRA
151 KFTSNIQVEL VQSLLPLVDS FEKTNVEVTL ETEKEQKIST SYQGIYKQLV
201 ETLKSLGVGV VETVGKPFDP VVHEAIAREE STEFKAGIVS HEVHRGFLLR
251 ERVLRPAAVK VSTGPGDQNT SSTTSEEPVE DTKEDAAV
```

Blast

Any recognized protein with high homology.

Spot 18. cp31BHv (nucleic acid-binding protein); gi|3550483; CAA11893; Score: 1081; [Hordeum vulgare subsp. vulgare].

```
  1 MATSAMTLAM SAATEASLFH PAFAAQHKLA PASASLPLIF SRAPLLRSTR
 51 PRVPLTPLVT SSDAAEAGLD WADAEEAEET VTEEEPVVAA SGGDAGYTAE
101 PPEEAKVYVG NLPYDVDSER LAQLFDQAGV VEVAEVIYNK ESGQSRGFGF
151 VTMSTIEEAD KAIETFNRYN ISGRLLNVNR AAQRGSRVER PPRQFASSFR
201 AYVGNLPWQA EDSRLVQMFS EHGEVVNATI VYDRETGRSR GFGFVTMASK
251 EDLDSAISAL DGQEMDGRPL RVNVAAERPQ RGF
```

Spot 19. cp31BHv (nucleic acid-binding protein); gi|3550483; CAA11893; Score: 1170; [Hordeum vulgare subsp. vulgare].

```
  1 MATSAMTLAM SAATEASLFH PAFAAQHKLA PASASLPLIF SRAPLLRSTR
 51 PRVPLTPLVT SSDAAEAGLD WADAEEAEET VTEEEPVVAA SGGDAGYTAE
101 PPEEAKVYVG NLPYDVDSER LAQLFDQAGV VEVAEVIYNK ESGQSRGFGF
151 VTMSTIEEAD KAIETFNRYN ISGRLLNVNR AAQRGSRVER PPRQFASSFR
201 AYVGNLPWQA EDSRLVQMFS EHGEVVNATI VYDRETGRSR GFGFVTMASK
251 EDLDSAISAL DGQEMDGRPL RVNVAAERPQ RGF
```

Spot 20. Predicted protein; gi|326504940; BAK06761;Score: 3114;[Hordeum vulgare subsp. vulgare].

```
  1 TGSQVVYSKY AGTEVEYNNS KHLIMKEDDI IGILESDDVK DMKPLNDRVL
 51 IKVAEASDKT EAGLILTETT KEKPSIGTVV AVGPGSLDEE GNRQPLSVSP
101 GSTVLYSKYA GGEFKGTDGT NYIVLRVSDV MAELS
```

Blast

Chaperonin; ACG41110.1; [Zea mays].

Spot 21. Predicted protein; gi|326490876; BAJ90105; Score: 348;[Hordeum vulgare subsp. vulgare].

```
  1 MALASTSPLA ATVARPKAPA SLTRCRSRRL QRISCQATTD RSGGGNASNT
 51 SPAPPRWRVA VSAALAAAVV VAMPAHADLN KYEADQRGEF GIGSAAQFGN
101 ADLKNTVHVN ENFRRANFTS ADMRESDFSG STFNGAYMEK AVAFRANFTG
151 ADLSDTLMDR MVLNEANLTN AVLSRTVLTR SDLGGATIEG ADFSDAVIDL
201 PQKLALCKYA SGTNPITGVS TRKSLGCGNS RRNAYGSPSS PLLSAPPPKL
251 LDRDGFCDEA SGLCDAK
```

Blast

Any recognized protein with high homology.

Spot 22. ES2A [gibberellic acid (GA3)inducible]; gi|929669; CAA55976; Score: 987; [Hordeum vulgare].

```
  1 MSSWLQEKSG EVVETTQVKA GEATKMASET GQSIQDRAVE AKDQTGSFLG
 51 EKSEAVTKSA SETTEAAKKM GGEAMGKVSE TVQAGQDRAV EGKDQTASFL
101 GEKTEAVKKT ATETADAAKE KSTEAAQHVQ DTAAEYTKDT PVAPKENVFQ
151 KAGGNIVGAA TDAKDAVMNT LGMGGDK
```

Spot 23. Predicted protein; gi|326504940;BAK06761;Score: 230;[Hordeum vulgare subsp. vulgare].

```
  1 TGSQVVYSKY AGTEVEYNNS KHLIMKEDDI IGILESDDVK DMKPLNDRVL
 51 IKVAEASDKT EAGLILTETT KEKPSIGTVV AVGPGSLDEE GNRQPLSVSP
101 GSTVLYSKYA GGEFKGTDGT NYIVLRVSDV MAELS
```

Blast

Chaperonin; ACG41110.1; [Zea mays].

Spot 24. Os02g0622400; gi|115447377; NP_001047468; Score: 113;[Oryza sativa Japonica Group].

```
  1 MAETVFTPSL EGMKHVKSES SVILTKPFLD VCKQILPVLD KFGAAMALVK
 51 SDIGGNITRL ENKYSSDPSK YEQLYSMVQE EVQNKTAKGS SSCTNGLLWL
101 TRAMDFLVEL FRNLLEHQDW TMSQACTDSY TKTLKKWHGW LASSSFTVAM
151 KLAPNREKFM EVISGTGDIK ADIEKFCTTF YPFLKENHDF LASVGLDDLK
201 AS
```

Blast

glycolipid transfer protein-like; BAD22518.1|;[Oryza sativa Japonica Group] >.

Spot 25. Unnamed protein product; gi|11587; CAA25265; Score: 708; [Hordeum vulgare].

```
  1 MSPQTETKAG VGFQAGVKDY KLTYYTPEYE TKDTDILAAF RVSPQPGVPP
 51 EEAGAAVAAE SSTGTWTTVW TDGLTSLDRY KGRCYHIEPV AGEDSQWICY
101 VAYPLDLFEE GSVTNMFTSI VGNVFGFKAL RALRLEDLRI PPTYSKTFQG
151 PPHGIQVERD KLNKYGRPLL GCTIKPKLGL SAKNYGRACY ECLRGGLDFT
201 KDDENVNSQP FMRWRDRFVF CAEAIYKSQA ETGEIKGHYL NATAGTCEEM
251 IKRAVFAREL GVPIVMHDYL TGGFTANTTL AHYCRDNGLL LHIHRAMHAV
301 IDRQKNHGMH FRVLAKALRM SGGDHIHSGT VVGKLEGERE MTLGFVDLLR
351 DDFIEKDRAR GIFFTQDWVS MPGVIPVASG GIHVWHMPAL TEIFGDDSVL
401 QFGGGTLGHP WGNAPGAAAN RVALEA
```

Blast

ribulose 1,5-bisphosphate carboxylase/oxygenase large subunit; YP_874661.1; Hordeum vulgare subsp.

Spot 26. Germin-like protein 1; gi|4239821; BAA74702; Score: 2249;[Oryza sativa].

```
  1 MAKAVMMLPV LLSFLLLPFS SMALTQDFCV ADLTCSDTPA GYPCKASVGA
 51 GDFAYHGLAA AGNTSNLIKA AVTPAFVGQF PGVNGLGISA ARLDIAVGGV
101 VPLHTHPAAS ELLFVTQGTV AAGFITSSSN TVYTRTLYAG DIMVFPQGLL
151 HYQYNAGQSA AVALVGFSGP NPGLQINDYA LFANNLPSAI VEKVTFLDDA
201 QVKKLKSVLG GSG
```

Spot 27. Predicted protein**;** gi|326500102; BAJ90886; Score: 161;[Hordeum vulgare subsp. vulgare].

```
  1 MSGVKKVADV AAKAGKAIDW DGMAKMLVSE EARKEFANLR RTFEDVNHQL
 51 QTKFSQEPQP IDWEYYRKGI GSKVVDMYKE AYDSIEIPKY VDTVTPRYKP
101 KFDALLVELK EAEKTSLKES ERIEKEIAEM KEMKKKISTM TADEYFAKHP
151 ELKKKFDDEM RNDYWGY
```

Blast

ATP synthase D chain, mitochondrial, putative; AAT40531.1; [Solanum demissum].

Spot 28. Eukaryotic translation initiation factor 5A1; gi|74048999; AAZ95171; Score: 447; [Triticum aestivum].

```
  1 MSDTDEHHFE SKADSGASKT YPQQAGAIRK GGHIVIKARP CKVVEVSTSK
 51 TGKHGHAKCH FVAIDIFNGK KLEDIVPSSH NCDVPHVDRQ DYQLIDITDD
101 GYVSLLTESG NTKDDLKLPT DDVLLGQIKT GFADGKDLIL SVMSAMGEEQ
151 ICAVKEIGGG K
```

Spot 29. RuBisCO large subunit-binding protein subunit beta, chloroplastic (60 kDa chaperonin subunit beta); gi|2493650; Q43831; Score: 1069; [Secale cereale].

```
  1 PQIVNDGVTV AREVELEDPV ENIGAKLVRQ AAAKTNDLAG DGTTTSVVLA
 51 QGLIAEGVKV IAAGANPVQI TRGIEKTAKA LVLELKKMSK EVEDSELADV
101 AAVSAGNNYE IGNMIAEAMS KVGRKGVVTL EEGRSSENNL YVVEGMQFER
151 GYISPYFVTD SEKMTTEYEN CKLLLVDKKI TNARDLINVL EEAIRGQYPI
201 LIIAEDIEQE ALATLVVNKL RGSLKICAIK APGFGERKTQ YLDDIAILTG
251 GTVIRDEVGL TLDKADNTVL GTAAKVVLTK ESTTIVGDGS TQEEVTKRVA
301 QIKNLIEAAE QDYEKEKLNE RIAKLAGGVA VIQVGAQTET ELKEKKLRVE
351 DALNATKAAV EEGIVVGGGC TLLRLAAKVD AIKDTLENDE QKVGAEIVRR
401 ALCYPLKLIA KNAGVNGSVV TEKVLSNDNF KFGYNAATGQ YEDLMAAGII
451 DPTKVVRCCL EHAASVAKTF LTSDVVVVEI KEPEAAPLAN PMDNSGFGY
```

Spot 30. Predicted protein; gi|326496140; BAJ90691; Score: 567;[Hordeum vulgare subsp. vulgare].

```
  1 MAAATSSFAT LAVARPAAAA QRALLAAKAP SSALSLRGVG RVASPALSVS
 51 LQTRARFVAS ASAEPYAPEL QSKVTNKVYF DINIGNPVGK NVGRIVIGLY
101 GDDVPQTVEN FRALCTGEKG FGYKGSSFHR VIKDFMIQGG DFDKGNGTGG
151 KSIYGRTFKD ENFQLVHTGP GVLSMANAGP NTNGSQFFIC TVKTPWLDGR
201 HVVFGQVLEG MDIVRMIESS ETDRGDRPKK KVVISECGEL PVV
```

Blast

cyclophilin-like protein; AAP44537.1; [Triticum aestivum].

Spot 31. Huntingtin interacting protein K; gi|195632082; ACG36699; Score: 142; [Zea mays].

```
  1 MGAAGDEKAA MAASAAAAGA GAAEGEGAVD SKDLQQQSKA LDKLTDHVED
 51 RQLDSSRVQS AMAALASSKE ADWNAMRLRE KELAAVKINP ADVEIIASEL
101 ELDKKIAERT LREHKGDAVA AVRFLLR
```
